# Isolation and characterization of lytic *Shigella* bacteriophages with rapid *in vitro* and *ex vivo* bactericidal activity

**DOI:** 10.3389/fcimb.2026.1837303

**Published:** 2026-06-16

**Authors:** Nida Shaheen, Maranda Stricklin, Martin Kordesch, Shaohua Wang

**Affiliations:** 1 Department of Biomedical Sciences, Ohio University Heritage College of Osteopathic Medicine, Ohio University, Athens, OH, United States; 2 Infectious and Tropical Disease Institute, Ohio University, Athens, OH, United States; 3 Department of Biological Sciences, Virginia Polytechnic and State University, Blacksburg, VA, United States; 4 Department of Physics and Astronomy, Ohio University, Athens, OH, United States

**Keywords:** antimicrobial alternatives, ex vivo fecal microbiota model, host range, lytic bacteriophages, multidrug resistance, phage therapy, Shigella spp.

## Abstract

**Introduction:**

The emergence of shigellosis caused by *Shigella* spp. poses a significant threat to global health, and the prevalence of multidrug-resistant (MDR) strains necessitates the development of effective antimicrobial therapies. Bacteriophages present a promising approach for both prevention and treatment of MDR infections.

**Methods:**

Ten lytic bacteriophages targeting *Shigella flexneri* 2457T were isolated from wastewater using the double-layer agar method. Phage morphology, lytic activity spectrum, latent period, burst size, bacterial killing efficiency, and efficacy under ex vivo microbiota conditions were evaluated. Transmission electron microscopy, one-step growth assays, bacterial killing assays, anaerobic fecal slurry co-culture experiments, and whole-genome sequencing of the representative phage PSW32 were performed.

**Results:**

Transmission electron microscopy revealed that these phages possessed icosahedral heads and contractile tails, consistent with myovirus-like morphology commonly observed among tailed bacteriophages within the class *Caudoviricetes*. Most phages exhibited broad lytic activity against *S. flexneri, S. sonnei*, and *S. dysenteriae*, with limited activity against *S. boydii* in spot assay. Among them, phages PSW32, PSW37, PSW38, and PSW40 exhibited lytic activity against the largest number of tested *Shigella* strains under the spot assay conditions. One-step growth assays revealed latent periods of 30-70 minutes and burst sizes of 40-267 PFU/cell, indicating efficient host infection activity. At multiplicities of infection (MOIs) ranging from 10 to 0.001, all phages efficiently suppressed the growth of *S. flexneri*. Based on their lytic kinetics and host range, PSW32 and PSW40 were further evaluated in an ex vivo microbiota model using anaerobic fecal slurry co-cultures. PSW32 reduced CFUs by 88% at 2 h and decreased bacterial levels to below the limit of detection (LOD) by 3 h. PSW40 reduced CFUs by 69% at 2 h and reduced bacterial levels to below the limit of detection (LOD) by 3 h. Genomic analysis of PSW32 identified a T4-like lytic architecture with no detectable genes linked to antimicrobial resistance or virulence, supporting its potential applicability for further evaluation.

**Discussion:**

These newly isolated lytic phages exhibited rapid bacterial activity under both *in vitro* and *ex vivo* microbiota-associated conditions and broad lytic activity against multiple *Shigella* species, Collectively, the findings support their potential for further investigation as phage-based strategies for controlling *Shigella* infections and contamination.

## 1 Introduction

*Shigella* is a Gram-negative, rod-shaped, non-motile, facultative anaerobic, non-spore-forming bacterium belonging to the family *Enterobacteriaceae* (Ranjbar and Abbas, 2019; Kotloff et al., 2018; Pakbin et al., 2023). It is the causative agent of shigellosis, also referred to as bacillary dysentery, an acute inflammatory diarrheal disease that remains a major public health challenge, particularly in developing and underdeveloped countries (Hendrick et al., 2025). Globally, *Shigella* represents a major public health burden and is associated with approximately 200 million cases and up to 650,000 deaths annually, with the highest incidence among children aged 1–3 years (Berihu et al., 2024). In the United States, around 450,000 shigellosis cases occur each year, with the majority being caused by *S. sonnei* (CDC, 2024). Transmission occurs primarily via the fecal-oral route, with as few as 10–100 bacteria sufficient to establish infection. Consequently, outbreaks are prevalent in regions with inadequate sanitation and hygiene, including areas affected by contaminated food and water, malnutrition, overcrowding, and limited public health awareness (CDC, 2024; Pakbin et al., 2021). In addition to person-to-person transmission, foodborne contamination serves as a significant route of infection. *Shigella* has been detected in vegetables, poultry, salads, fruits, and dairy products, contributing to numerous outbreaks (Pakbin et al., 2022). Globally, foodborne *Shigella* infection accounts for an estimated 1–3 million disability-adjusted life years (DALYs) annually (Havelaar et al., 2015).

The genus *Shigella* includes four pathogenic species: *S. dysenteriae, S. flexneri, S. boydii, and S. sonnei*, all of which are implicated in the development of shigellosis (Kotloff et al., 1999; Yang et al., 2018). *S. flexneri* and *S. dysenteriae* predominate in low- and middle-income countries, whereas *S. sonnei* is more common in industrialized regions (Hendrick et al., 2025; Zaidi and Estrada-García, 2014). *S. flexneri* can be further divided into 23 serotypes based on antigenic variation in the lipopolysaccharide O-antigen, with serotype conversion frequently mediated by temperate bacteriophages (Sadredinamin et al., 2023). Other *Shigella* species display a wide range of serotypes. Specifically, *S. dysenteriae*, and *S. boydii* each comprise approximately 15 and 19 serotypes, respectively. In contrast, *S. sonnei* is unique, with only one serotype, although it can be further divided into various genotypes and lineages (Kotloff et al., 2018).

Antibiotics remain the cornerstone of shigellosis treatment, with the World Health Organization (WHO) currently recommending fluoroquinolones (e.g., ciprofloxacin), pivmecillinam, azithromycin, and third-generation cephalosporins such as ceftriaxone (Azmi et al., 2014; Williams and James A., 2018). However, the widespread and inappropriate use of antibiotics has accelerated the emergence of multidrug-resistant (MDR) *Shigella* strains (Puzari et al., 2018). Increasing resistance has been reported to several first-line therapies recommended by the WHO, including fluoroquinolones such as ciprofloxacin, macrolides such as azithromycin, and third-generation cephalosporins such as ceftriaxone. This resistance presents significant challenges for the clinical management of shigellosis (Health Alert Network (HAN), 2025; Bowen et al., 2016; Asad et al., 2024). This adaptability is largely driven by mobile genetic elements, such as plasmids, transposons, insertion sequences, and genomic islands, which promote horizontal gene transfer and the rapid dissemination of resistance genes (Ranjbar and Abbas, 2019; Asad et al., 2024). Recent surveillance studies underscore the accelerating spread of extensively drug-resistant (XDR) *Shigella*. From 2005 to 2021, the monitoring of 7,121 *Shigella* (*S.*) *sonnei* isolates in France revealed a significant increase in the number of strains resistant to ciprofloxacin, third-generation cephalosporins, and azithromycin since 2015, indicating the rapid emergence of extensively drug-resistant (XDR) strains (Lefèvre et al., 2023). Similarly, in the United Kingdom, an outbreak of sexually transmitted XDR *S. sonnei* was reported among men who have sex with men (MSM), underscoring ongoing transmission in affluent areas (Charles et al., 2022). In the United States, XDR *Shigella* resistant to both empiric and alternative antibiotics have also been reported, with prevalence increasing from 0% in 2015 to 5% in 2022 (Control, Centers for Disease, Prevention (CDC), 2023). Recent genomic studies in the U.S. further identified multidrug-resistant (MDR) outbreaks, including an *S. flexneri* 2a cluster in New Mexico (affecting humans and non-human primates) and a 2024 *S. sonnei* outbreak in California, highlighting ongoing domestic transmission (Domman et al., 2024). Consequently, the WHO has identified *Shigella* as a priority pathogen requiring the development of novel non-antibiotic antimicrobial strategies (Clarkson et al., 2024). These findings highlight the urgent need for innovative alternatives that can effectively control *Shigella* infection while reducing dependence on conventional antibiotics.

Bacteriophages, viruses that specifically infect bacteria, represent a promising alternative to antibiotics for the treatment of shigellosis. They are ubiquitous in ecosystems inhabited by bacteria (Clokie et al., 2011; Youssef et al., 2025), replicating within host cells, hijacking cellular machinery, and subsequently releasing mature phage particles through host cell lysis (Ofir and Rotem, 2018; Valencia-Toxqui and Jolene, 2024). *Shigella* phages were initially discovered in the early 20th century by Twort (Twort, 1915) and Felix d’Herelle (Fruciano and Shawna, 2007; Herelle, 1931), who later demonstrated their therapeutic potential by successfully treating dysentery caused by *S. dysenteriae* (Zhang et al., 2013). Since then, over 78 lytic *Shigella* phages have been isolated from environmental sources, demonstrating potent bactericidal activity (Subramanian et al., 2020). Phages are abundant, highly host-specific, and generally regarded as safe for human application (Peng et al., 2019; Palma and Bowen, 2024). However, candidate therapeutic phages must be thoroughly evaluated for host range, burst size, stability, and genomic safety (Merabishvili et al., 2009). Moreover, despite the extensive documentation of *Shigella* phages, the rise of MDR and XDR strains, along with frequent outbreaks, continues to present a significant clinical challenge. Previous research has mainly focused on phage activity in axenic cultures, with limited evaluation under anaerobic conditions that better reflect the complexity of the gut environment or across a broader, clinically relevant range of multiplicities of infection (MOIs). Furthermore, only a few studies have assessed their efficacy against contemporary clinical MDR isolates, highlighting a critical gap in translational research.

In this study, we isolated, purified, and morphologically characterized novel lytic *Shigella* phages, and performed whole-genome sequencing and analysis of a representative isolate (PSW32). In addition to general characterization, selected phages were further validated using an anaerobic fecal slurry model representing *ex vivo* microbiota conditions. These phages effectively reduced bacterial population within this complex microbial community and exhibited broad coverage across *Shigella* species. While no lytic activity was observed against tested non-target strains *in vitro*, the broader impact on microbiota composition was not directly assessed. By integrating laboratory characterization with ex vivo microbiota-associated validation, this study supports further investigation of these phages for *Shigella* biocontrol and related phage-based applications.

## 2 Materials and methods

### 2.1 Isolation, purification, and amplification of bacteriophages

The isolation of *S. flexneri*-specific bacteriophages was performed following established protocols (Mallick et al., 2021; Meidaninikjeh et al., 2024), with modifications to accommodate the *S. flexneri* 2457T host. As part of the campus wastewater surveillance initiative, wastewater samples were collected from multiple sites at Ohio University and the Athens City County Wastewater Treatment facilities. Samples were first centrifuged (10,000 rpm, 10 min) to remove large particulate matter. The resulting supernatants were filtered through a 0.22 µm syringe filters to eliminate residual debris. To detect the presence of *Shigella* phages, 10 µL of each filtrate was spotted onto a lawn of *S. flexneri* 2457T using the double-layer agar method (Adams, 1959). The appearance of clear zones (plaques) indicated lytic activity against the host bacterium. To purify the phage, individual plaques were picked and subjected to two further successive rounds of plaque isolation. The purified phages were then enriched by overnight co-culture with *S. flexneri* 2457T (OD_600_ = 0.2) at 37 °C. Following incubation, bacterial cells were pelleted by centrifugation at 9000×g for 10 min, and the supernatant containing phage particles was filtered through 0.22 µm membrane. The resulting phage suspension was stored at 4 °C for subsequent experiments. Phage titration was performed after each enrichment cycle using a double-layer agar assay.

### 2.2 Transmission electron microscopy

The morphological characteristics of the bacteriophages were examined using transmission electron microscopy (TEM) following the procedure described by Meidaninikjeh et al. (2024). A double-layer agar plate exhibiting a high plaque density (≥10^9^ PFU/mL) was selected. Sterile deionized water (5 mL) was added to the plate, followed by gentle agitation at 70 rpm for six hours at room temperature, and then incubated overnight at 4 °C. The resulting phage suspension was centrifuged at 4000×g for 20 min, and the supernatant was filtered through a 0.22 µm membrane. The purified phage filtrate was negatively stained with uranyl acetate (2%) and prepared for TEM analysis. Electron micrographs of isolated phage particles were captured using a JEOL transmission electron microscope operated under appropriate accelerating voltage conditions. Phage morphology, including head shape and tail structure, was examined, and virion dimensions were measured to determine average size parameters.

### 2.3 Host range determination

The host range of the isolated bacteriophages was evaluated using spot assays against a panel of clinical isolates and reference bacterial strains, including *S. flexneri, S. dysenteriae, S. sonnei, S. boydii*, *Escherichia* (*E.*) *coli*, and *Klebsiella* (*K.*) *pneumoniae* strains. Among these, we obtained five clinical *Shigella* strains (2 *S. flexneri* and 3 *S. sonnei*) from Brigham and Women’s Hospital, most of which were multidrug-resistant (MDR) isolates (Worley et al., 2021) as well as an additional 61 *Shigella* strains (21 *S. flexneri*, 20 *S. sonnei*, 12 *S. boydii*, and 7 *S. dysenteriae*) from the San Diego County Public Health Laboratory. As *E. coli* and *Klebsiella* are closely related to *Shigella*, we also included our laboratory-stock uropathogenic *E. coli* strain UPEC-CET073, along with three recently identified *K. pneumoniae* isolates (8RL1 pink, A#7, and 8RL2; NCBI accession numbers PX671225, PX671227, and PX671226, respectively). A complete list of bacterial strains used in this study and their sources is provided in **Supplementary Table 3**.

Each bacterial strain was grown overnight at 37 °C in Luria-Broth (LB) broth. All spot assays were performed in at least two independent experiments as previously described (Jun et al., 2013). Briefly, each bacterial culture (1% inoculum) was mixed with soft agar (0.7%), 50 µL 0.1M MgSO_4_, and 10 µL of 1M CaCl_2_, and the mixture was overlaid onto LB agar plates to obtain a double layer. Thereafter, 10 µL of phage suspension was spotted onto the surface of the solidified double-layer agar. Plates were incubated overnight at 37 °C, and lytic activity was determined by the formation of clear plaques or visible zones of lysis. Strains exhibiting distinct lysis zones were classified as susceptible, whereas those without detectable lysis were considered resistant.

In addition, eleven probiotic strains (*Bifidobacterium pseudocatenulatum, B. breve, B. pseudolongum, B. longum, B. bifidum, Lactobacillus plantarum, L. rhamnosus, L. pantheris, L. sakei, Streptococcus salivarius, S. thermophilus*; NCBI accession numbers PQ454214, PQ454215, PQ454216, PQ454217, PQ454218, PQ454208, PQ454209, PQ454210, PQ454211, PQ454212 and, PQ454213 respectively) that had been previously isolated and characterized in our laboratory (Gurung et al., 2025) were included in the host range analysis to further assess phage specificity and determine whether the phage exhibited lytic activity against beneficial gut-associated probiotics. Although these Gram-positive bacteria are not anticipated to serve as hosts for bacteriophages targeting *Shigella*, their inclusion provided an initial assessment of phage specificity toward beneficial gut-associated bacteria.

### 2.4 One-step growth analysis

A one-step growth assay was performed in two independent experiments with each time point analyzed in triplicate, following previously described methods with minor modifications (Choudhary et al., 2024). Briefly, *S. flexneri* 2457T was cultured to an optical density (OD_600_) of 0.2, which corresponded to approximately 1.0 × 10^8^ CFU/mL. Two hundred microliters of the bacterial culture were harvested by centrifugation at 8,000×g for 5 min at room temperature, re-suspended in SM buffer (Wang et al., 2010), and mixed with phage lysate at a multiplicity of infection (MOI) of 0.01. To reduce variability in infection timing and minimize secondary adsorption events, the phage-host mixture was incubated at 37 °C for 15 min to allow phage adsorption prior to removal of unadsorbed phages by centrifugation. This adsorption period was implemented across all isolates as a standardized experimental condition for comparative one-step growth analyses. Following incubation, the mixture was centrifuged at 12,000×g for 2 min to remove unadsorbed phages, and the resulting pellet was resuspended in 10 mL of LB broth supplemented with MgSO_4_ (1 mM) and CaCl_2_ (2 mM). Removal of free phage particles prior to incubation effectively minimized secondary adsorption events, thereby allowing characterization of phage replication kinetics under controlled adsorption conditions. The culture was incubated at 37 °C with shaking at 200 rpm, and samples were collected every 10–20 min interval to determine phage titers using the double-layer agar method. The latent period was defined as the interval between initial infection and the first observed increase in phage titer (Khan Mirzaei and Anders, 2015). Burst size was calculated as the ratio of liberated phage particles to the number of initially infected bacterial cells during the latent period (Yang et al., 2020).

### 2.5 Killing efficiency

The optimal multiplicity of infection (MOI) for each phage was determined as described by Zhong et al (Zhong et al., 2024). An overnight culture of *S. flexneri* 2457T was subcultured into fresh LB broth and incubated at 37 °C until it reached an optical density at 600 nm (OD_600_) of 0.2. Five MOIs (0.001, 0.01, 0.1, 1, and 10) were prepared by serially diluting phage stocks. Bacterial cultures were mixed with diluted phages and LB broth supplemented with MgSO_4_ (1 mM) and CaCl_2_ (2 mM) in 96-well plates to a final volume of 200 µL per well. Wells containing only medium or bacteria served as negative and positive controls, respectively. Each MOI was tested in triplicate. Plates were incubated at 37 °C, and OD_600_ was measured every 15 min for 7 h using a Synergy H1 microplate reader (BioTek). Results are presented as mean ± standard deviation (SD).

### 2.6 Efficiency under *ex vivo* microbiota conditions

Among the identified phages, PSW32 and PSW40 were selected for evaluation under ex vivo microbiota conditions based on their broad spot-assay-based lytic activity across all four *Shigella* species, reproducible propagation characteristics, and distinct replication dynamics observed in one-step growth and killing assays. PSW32 exhibited rapid lytic activity and a high burst size, while PSW40 demonstrated more moderate replication traits but maintained broad lytic activity under the tested spot assay conditions. By including these two candidates with different performance profiles, we sought to determine whether their lytic efficacy was maintained under ex vivo microbiota conditions. This assay was adapted from Ahmadi et al. (2019). An anaerobic dilution solution (5 g NaCl, 2 g glucose, and 0.3 g cysteine-HCl per liter) was pre-reduced in an anaerobic chamber prior to use. For fecal slurry preparation, 5 g of pooled fecal samples were suspended in 50 mL of the anaerobic diluent, homogenized for 15 min, and filtered through four layers of sterile cheesecloth. The filtrate was used immediately as the inoculum. For each treatment and control condition, 250 µL of *S. flexneri* 2457T (OD_600_ = 0.2), 250 µL of 0.1M MgSO_4_, 50 µL of 1M CaCl_2_, and 250 µL of filtered fecal inoculum were added to 25 mL of SM medium (Schaedler broth supplemented with sheep blood and hemin) (Średnicka et al., 2023). Phages were added at an MOI of 0.01. Cultures were incubated anaerobically at 37 °C. Aliquots (1.4 mL) were collected at 0, 1, 2, and 3 h, serially diluted (10^-5^ – 10^-6^), and 100 µL of each dilution was spread on xylose lysine deoxycholate (XLD) agar for *Shigella* enumeration. All experiments were conducted in duplicate, and CFU values are reported as mean ± standard deviation (SD). The limit of detection (LOD) was 1.0×10^6^ CFU/mL, based on the lowest dilution plated (10^-5^) and a plating volume of 100µL. The remaining portions were centrifuged at 13,000×g for 10 min at 4 °C, and the supernatants were collected for phage titration.

### 2.7 Whole genome sequencing

Among the ten isolated *Shigella* phages, PSW32 was selected for comprehensive genome sequencing as a representative isolate owing to its potent lytic activity, rapid bacterial clearance at low MOI, broad spot assay-based lytic activity, and consistent high-titer propagation, indicating its potential as a promising obligately lytic phage. These features identified PSW32 as a promising candidate for detailed genomic characterization and future *in vivo* studies. Therefore, comprehensive genomic analysis was performed for this phage. For genomic DNA extraction, a high-titer, filter-sterilized phage lysate (10¹^0^ PFU/mL) was prepared. The lysate was treated with DNase I and RNase A to remove non-encapsulated bacterial DNA and RNA. Briefly, nuclease treatment was employed to eliminate any residual host nucleic acids before capsid disruption. Subsequently, viral capsids were lysed according to the manufacturer’s instructions to release the encapsulated phage DNA before column-based purification. Viral DNA purification was then performed using a Phage DNA Isolation Kit (“Phage DNA Isolation Kit | Norgen Biotek Corp., Thorold, ON, Canada”), according to the manufacturer’s instructions. DNA quality and concentration were evaluated prior to sequencing. Purified genomic DNA was submitted to SeqCenter (Pittsburgh, PA) for whole-genome sequencing on an Illumina platform. Following quality filtering, reads were assembled *de novo* into a single circular double-stranded DNA contig representing the complete double-stranded DNA genome. Genome annotation was conducted using Prokka and Bakta to predict open reading frames (ORFs) and assign putative functions. Comparative sequence analyses were performed using BLASTn and BLASTp against the NCBI database to evaluate nucleotide similarity and relatedness to reference phages. A comprehensive phylogenetic analysis of the entire genome was conducted using the VICTOR (Virus Classification and Tree Building Online Resource) platform, which utilizes genome-wide nucleotide sequence comparisons, to determine the phylogenetic position of PSW32 in relation to closely related reference bacteriophages. Circular genome visualization and feature mapping were generated using Proksee (https://proksee.ca). Antimicrobial resistance genes were screened using the CARD Resistance Gene Identifier (RGI) implemented within Proksee under default parameters. Additionally, potential virulence-related genes were evaluated through database comparisons and functional annotation on the same platform.

### 2.8 Statistical analysis

Experiments were conducted in duplicate or triplicate, as specified in the relevant Methods sections and figure legends. Data are presented as mean ± standard deviation (SD). Statistical analyses were performed using GraphPad Prism 10 (GraphPad Software, San Diego, CA, USA). Student’s t-test was used for exploratory comparative analysis between phage-treated and untreated control groups. Due to the limited biological replicate numbers in certain experiments, a comprehensive assessment of normality assumptions was not feasible; therefore, the statistical conclusions should be considered as preliminary. A P-value of less than 0.05 was considered statistically significant.

## 3 Results

### 3.1 Isolation and morphology of phages

**Figure 1:**
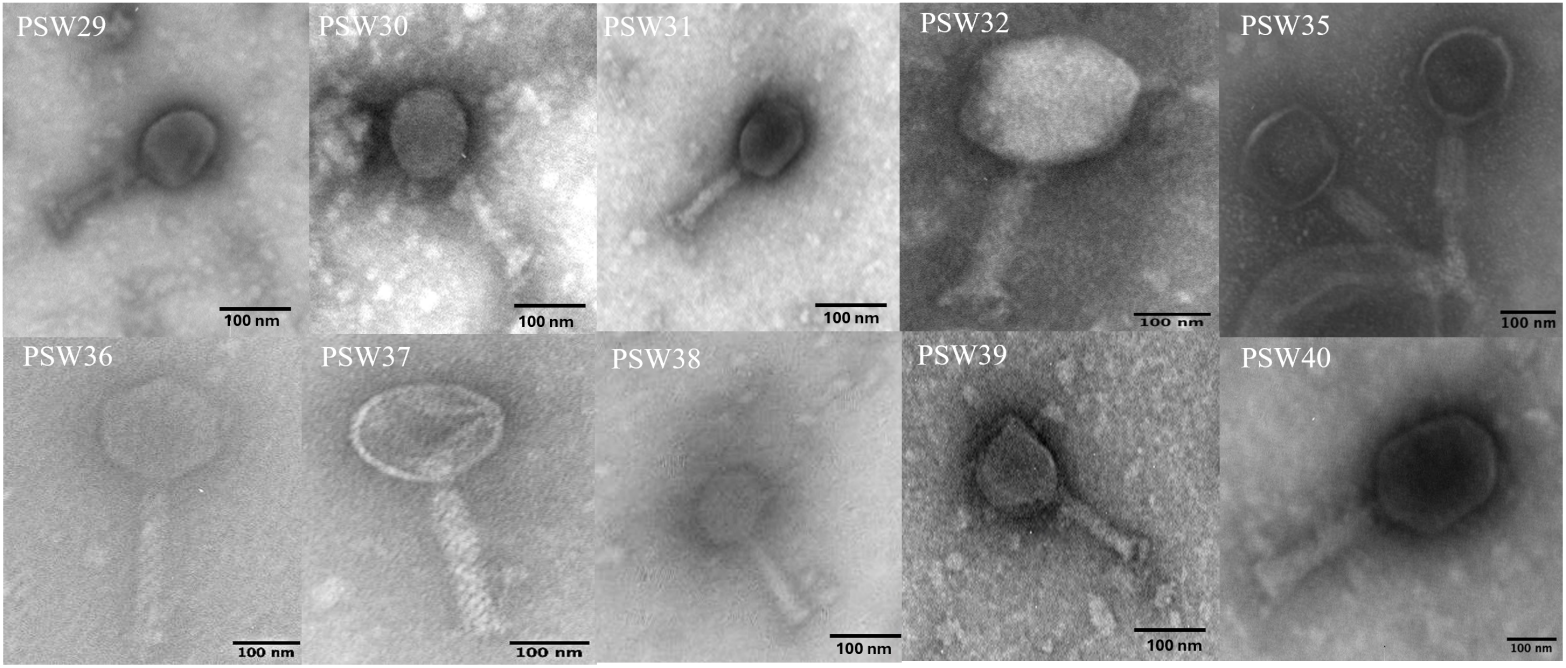
Transmission electron micrographs of isolated lytic *Shigella* bacteriophages negatively stained with 2% uranyl acetate. The phages displayed icosahedral heads and contractile tails, consistent with myovirus-like morphology observed among tailed bacteriophages. Representative images are presented at a unified scale to facilitate direct morphological comparison among isolates. Scale bars = 100 nm.

A total of ten phages capable of infecting the *S. flexneri* 2457T isolate were successfully isolated. All phages formed clear, round plaques with well-defined borders, indicative of robust lytic activity. Transmission electron microscopy (TEM) revealed that all isolated phages possessed icosahedral heads and contractile tails (**Figure 1**), consistent with myovirus-like morphology commonly observed among tailed bacteriophages within the class *Caudoviricetes* (Ackermann, 2007; Mallick et al., 2021). Because whole-genome sequencing was performed only for PSW32, formal taxonomic assignment was not attempted for the remaining phages. Although the phages exhibited generally similar morphologies, variations were observed in head dimensions and tail lengths, which ranged from 42.7–178 nm and 53.1–188 nm, respectively. Detailed morphological measurements for each phage are provided in **Supplementary Table 1**.

### 3.2 Host range determination

The lytic spectrum of the *Shigella* phages was preliminarily evaluated using spot assays, in which purified phage suspensions were applied to bacterial lawns of clinically isolated *S. flexneri* (n=24)*, S. sonnei* (n=23), *S. dysenteriae* (n=8), and *S. boydii* (n=12) strains, as well as additional bacterial species, including *E. coli* (n=1) and *K. pneumoniae* (n=3). Spot assays offer qualitative insights into lytic activity and may also reflect lysis-from-without effects. These findings are interpreted as indicative of potential host vulnerability rather than conclusive evidence of productive infection. As summarized in **Table 1**, all ten phages formed clear lytic zones against all 24 *S. flexneri* and 20 of the 23 *S. sonnei* strains, with the exception that PSW35 and PSW36 failed to show lytic activity against two and one *S. sonnei* strains, respectively. Five phages (PSW32, PSW35, PSW37, PSW38, and PSW40) produced clear lysis zones against all eight *S. dysenteriae* strains, whereas the remaining five phages were unable to infect six of them. All phages were able to lyse one *S. boydii* strain (*S. boydii* SDCPHL7), while seven phages (PSW32, PSW35, PSW36, PSW37, PSW38, PSW39, and PSW40) additionally infected another strain (*S. boydii* SDCPHL5); none exhibited activity against the remaining ten *S. boydii* strains. Among the non-*Shigella* species, the phages exhibited lytic activity against the tested uropathogenic *E. coli* UPEC-CET073 but did not produce lysis zones against *K. pneumoniae*. The results revealed no lytic activity against any of the probiotic strains tested, thereby supporting the apparent specificity of these isolated bacteriophages toward *Shigella* spp.

**Table 1:** Host range of isolated lytic *Shigella* phages.

Bacterial strains	PSW29	PSW30	PSW31	PSW32	PSW35	PSW36	PSW37	PSW38	PSW39	PSW40
*S. flexneri* 2457T	**+**	**+**	**+**	**+**	**+**	**+**	**+**	**+**	**+**	**+**
*S. flexneri* 0078-8604	**+**	**+**	**+**	**+**	**+**	**+**	**+**	**+**	**+**	**+**
*S. flexneri* 0091-4505	**+**	**+**	**+**	**+**	**+**	**+**	**+**	**+**	**+**	**+**
*S. flexneri* SDCPHL 21-41*	**+**	**+**	**+**	**+**	**+**	**+**	**+**	**+**	**+**	**+**
*S. sonnei* 0078-1844	**+**	**+**	**+**	**+**	**+**	**+**	**+**	**+**	**+**	**+**
*S. sonnei* 0079-2204	**+**	**+**	**+**	**+**	**+**	**+**	**+**	**+**	**+**	**+**
*S. sonnei* 0070-1115	**+**	**+**	**+**	**+**	**+**	**+**	**+**	**+**	**+**	**+**
*S. sonnei* SDCPHL 42-51*	**+**	**+**	**+**	**+**	**+**	**+**	**+**	**+**	**+**	**+**
*S. sonnei* SDCPHL 52	**+**	**+**	**+**	**+**	**+**	**-**	**+**	**+**	**+**	**+**
*S. sonnei* SDCPHL 55	**+**	**+**	**+**	**+**	**-**	**+**	**+**	**+**	**+**	**+**
*S. sonnei* SDCPHL 61	**+**	**+**	**+**	**+**	**-**	**+**	**+**	**+**	**+**	**+**
*S. sonnei* SDCPHL 53,54,56-60*	**+**	**+**	**+**	**+**	**+**	**+**	**+**	**+**	**+**	**+**
*S. dysenteriae* O-4576S1-G	**+**	**+**	**+**	**+**	**+**	**-**	**+**	**+**	**+**	**+**
*S. dysenteriae* SDCPHL 13-15, 18-19*	**-**	**-**	**-**	**+**	**+**	**+**	**+**	**+**	**-**	**+**
*S. dysenteriae* SDCPHL 16	**-**	**-**	**+**	**+**	**+**	**+**	**+**	**+**	**+**	**+**
*S. dysenteriae* SDCPHL 17	**+**	**+**	**+**	**+**	**+**	**+**	**+**	**+**	**+**	**+**
*S. boydii* SDCPHL 1-4, 6*	**-**	**-**	**-**	**-**	**-**	**-**	**-**	**-**	**-**	**-**
*S. boydii* SDCPHL 5	**-**	**-**	**-**	**+**	**+**	**+**	**+**	**+**	**+**	**+**
*S. boydii* SDCPHL 7	**+**	**+**	**+**	**+**	**+**	**+**	**+**	**+**	**+**	**+**
*S. boydii* SDCPHL 8-12*	**-**	**-**	**-**	**-**	**-**	**-**	**-**	**-**	**-**	**-**
*E. coli* UPEC-CET073	**+**	**+**	**-**	**-**	**-**	**+**	**-**	**-**	**-**	**-**
*Klebsiella* 8rl1pink, a#7, 8rl2	**-**	**-**	**-**	**-**	**-**	**-**	**-**	**-**	**-**	**-**

* S. flexneri SDCPHL 21–41 include strains 21, 22, 23, 24, 25, 26, 27, 28, 29, 30, 31, 32, 33, 34, 35, 36, 37, 38, 39, 40, 41, *S. sonnei* SDCPHL 42–51 include strains 42, 43, 44, 45, 46, 47, 48, 49, 50, 51, *S. sonnei* SDCPHL 53,54,56, 57, 58, 59, 60, *S. dysenteriae* SDCPHL 13, 14, 15, 18, 19, *S. boydii* SDCPHL 1, 2, 3, 4, 6, *S. boydii* SDCPHL 8, 9,10, 11, 12.

Based on the spot assay results, PSW32, PSW37, PSW38, and PSW40 demonstrated lytic activity against the broadest range of strains across all four *Shigella* species. PSW36 and PSW39 showed moderate activity, whereas PSW29 and PSW31 exhibited comparatively narrower lytic spectra. Notably, *S. flexneri and S. sonnei* were the most consistently susceptible species, as indicated by clear lysis zones by all phages tested. Collectively, these results reveal diverse patterns of lytic activity among the isolated phages in spot assays, underscoring their potential applicability against *Shigella* spp., while warranting further quantitative validation (e.g., efficiency of plating) to confirm productive infection across susceptible strains.

### 3.3 One-step growth analysis

**Figure 2:**
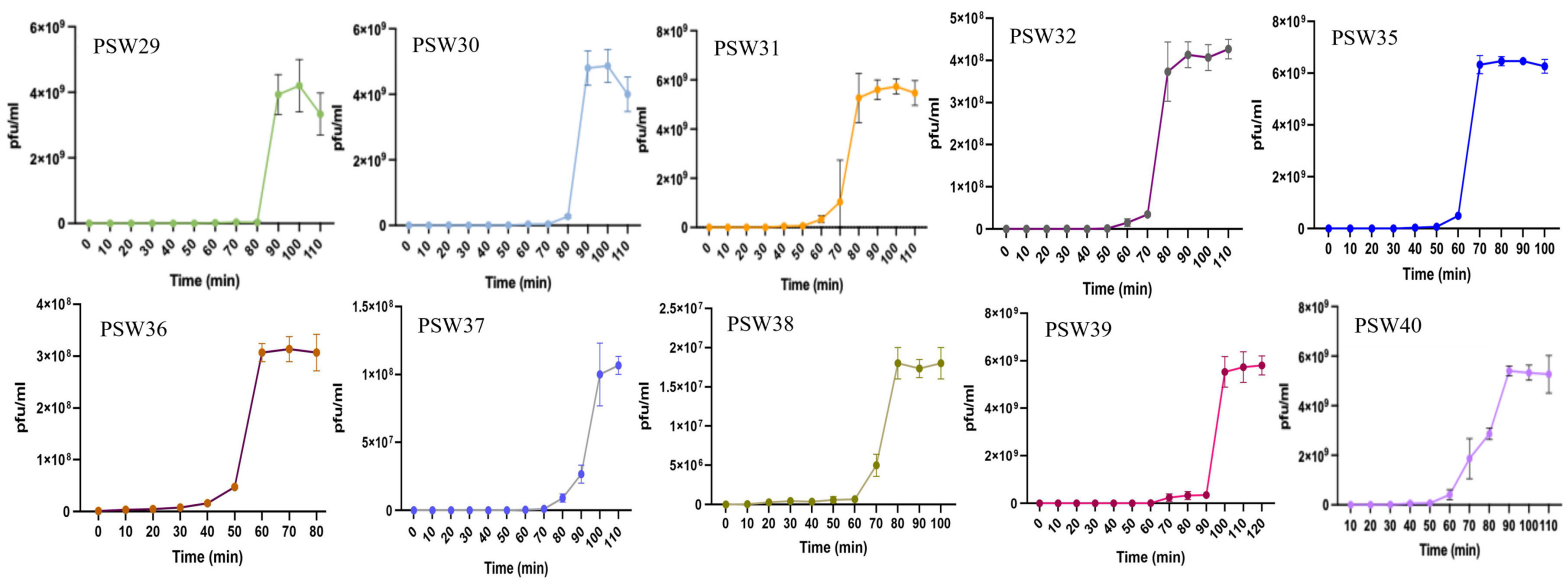
One-step growth curves of isolated lytic *Shigella* phages infecting *S. flexneri* 2457T. Phage replication dynamics were evaluated using a one-step growth assay at a multiplicity of infection (MOI) of 0.01. Samples were collected at 10–20 min intervals, and phage titers were measured over time are expressed as PFU/mL. Burst size values were determined using the double-layer agar method, calculated separately and are reported as PFU/cell in **Supplementary Table 2**. Error bars represent standard deviations (SD), and the data represent mean ± standard deviation (SD) of three independent determinations.

One-step growth experiments revealed variations in the latent period and burst size among the ten isolated *Shigella* phages (**Figure 2**; **Supplementary Table 2**). Latent periods ranged from 30 to 70 minutes. PSW36 and PSW29 exhibited the shortest latent periods (30 and 40 minutes, respectively), suggesting relatively rapid replication dynamics. In contrast, PSW30 and PSW37 showed the longest latent periods (70 minutes), suggesting slower replication kinetics. Burst sizes also varied substantially, ranging from 27 to 267 PFU/cell. PSW40 exhibited a latent period of 50 min and achieved a burst size of 81 PFU per infected cell, indicating a moderate replication efficiency relative to other isolates. PSW32 and PSW39 exhibited the largest burst sizes (259 and 267 PFU/cell, respectively), indicating highly efficient progeny production. In contrast, PSW38 and PSW36 showed the smallest burst sizes (27 and 40 PFU/cell), suggesting lower replication efficiency or potential host-related constraints. Overall, these results demonstrate diverse replication dynamics among the isolated *Shigella* phages. Notably, PSW32 and PSW39 combined short-to-moderate latent periods with high burst sizes, whereas PSW40 exhibited moderate replication features with a balanced latent period and burst size. Phage selection for further evaluation was based on overall lytic performance rather than burst size alone.

### 3.4 Killing efficiency

**Figure 3:**
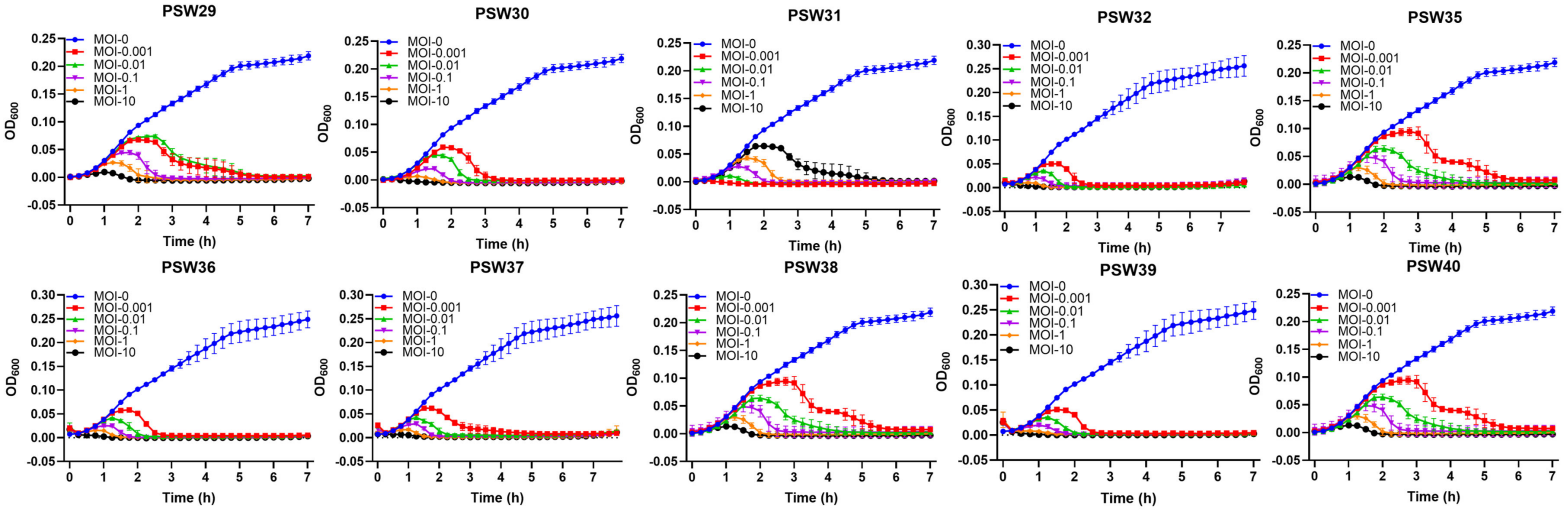
MOI-dependent lytic kinetics of isolated bacteriophages against *S. flexneri*, monitored by measuring bacterial growth (OD_600_) over time at different multiplicities of infection (MOI = 0, 0.001, 0.01, 0.1, 1, and 10). Error bars indicate standard deviation (SD). Data represent mean ± standard deviation (SD) of three independent experiments.

The lytic efficacy of all isolated phages against *Shigella* was assessed at different multiplicities of infection (MOIs: 0.001, 0.01, 0.1, 1, and 10) (**Figure 3**). Remarkably, even the lowest MOI (0.001) resulted in near-complete suppression of bacterial growth based on OD measurements within 2–5 h, demonstrating robust lytic activity across the phage collection. Phages PSW32, PSW36, and PSW39 exhibited the most rapid lytic activity, resulting in near-complete suppression of bacterial growth based on OD measurements within 2.5 h at all tested MOIs. Phages PSW30 and PSW37 exhibited a modest delay, achieving marked suppression of bacterial growth within approximately 3 h. In contrast, phages PSW29, PSW31, PSW35, PSW38, and PSW40 required slightly longer (~5 h) to achieve marked suppression of bacterial growth. Overall, these findings indicate strong lytic activity of all tested phages against *S. flexneri*, with minor variations in the time required for complete lysis. Notably, the ability of these phages to reduce bacterial growth even at very low MOIs highlights their robust replication capacity and strong antibacterial activity and support their further investigation under physiologically relevant conditions. It is important to emphasize that these findings are based on optical density measurements, which do not distinguish between viable and non-viable cells. Therefore, assays utilizing colony-forming units (CFU) are necessary to confirm bacterial reduction and assess the potential for regrowth or development of resistance.

### 3.5 Efficiency under ex vivo microbiota conditions

**Figure 4:**
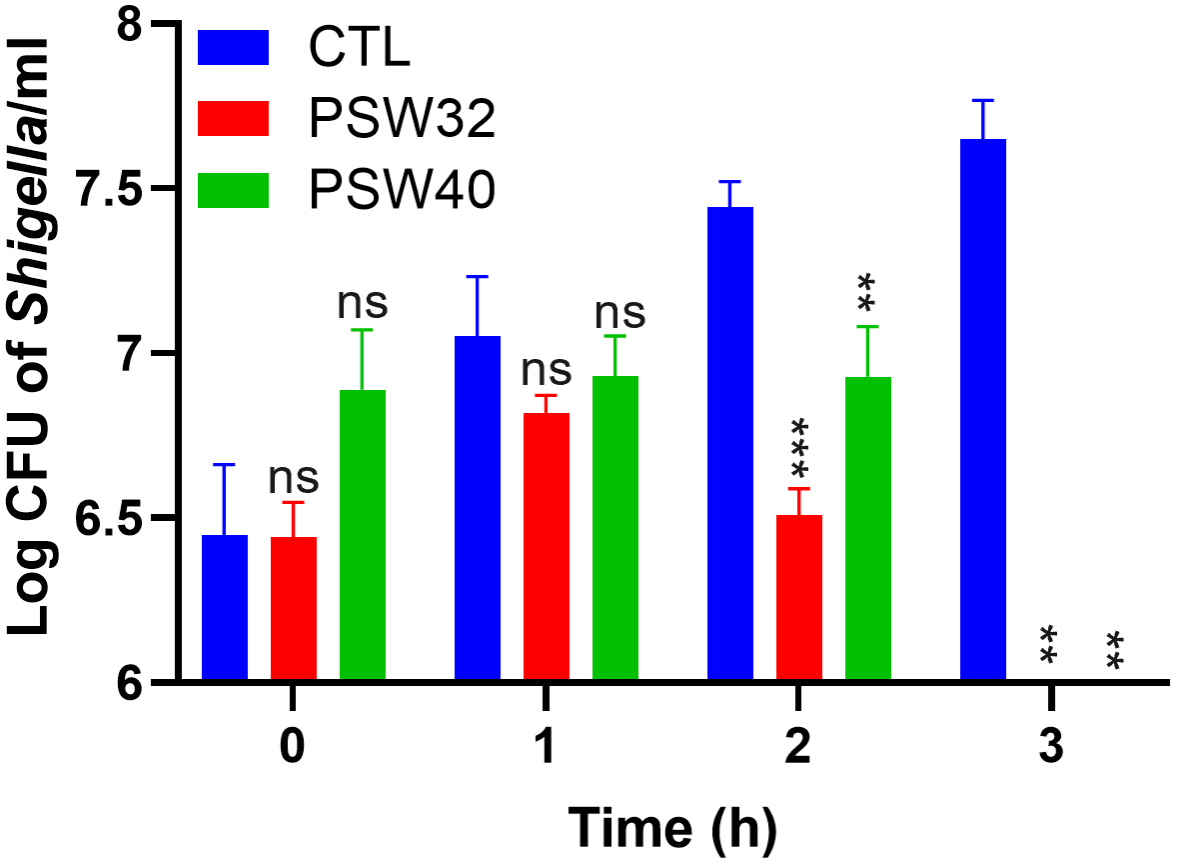
Efficiency of phages PSW32 and PSW40 against *S. flexneri* under ex vivo microbiota conditions. Bacterial concentrations are presented as (log10 CFU/mL) and were monitored over time in comparison with an untreated control (CTL). Error bars indicate standard deviations (SD). Data represent mean ± standard deviation (SD) of two independent experiments. Ns, not significant; **P < 0.01 and ***P < 0.001 indicate statistically significant differences relative to the control based on comparative statistical analysis using Student’s *t*-test.

Given the complex environment encountered during phage therapy for shigellosis, we further evaluated the efficacy of PSW32 and PSW40, both of which exhibit broad spot assay-based lytic activity but differ in killing efficacy and burst size, in eliminating the pathogen under ex vivo microbiota conditions using anaerobic fecal slurry cultures. Specifically, PSW32 displayed a high burst rate and rapid lytic activity, whereas PSW40 exhibited moderate productivity (**Figure 4**). Both phages significantly suppressed the growth of *S. flexneri* 2457T. In the phage-free control, CFU counts increased nearly tenfold over 2 h (from 2.8×10^6^ to 2.77×10^7^). In contrast, PSW32 treatment resulted in a slight initial increase (6.6×10^6^ at 1 h), followed by a ~51% decline to 3.23×10^6^ at 2 h (corresponding to an approximately 88% reduction relative to the control). PSW40 also maintained bacterial levels near baseline (7.73 × 10^6^ at 1 h and 8.50 × 10^6^ at 2 h), corresponding to a 69% reduction compared with the control at 2 h. By 3 h, both phages reduced bacterial counts to below the practical limit of detection [LOD] of 1.0×10^6^ CFU/mL under the plating conditions used. These findings demonstrate that PSW32 and PSW40 are both capable of substantial suppression of *S. flexneri* growth in a complex gut microbial environment, achieving rapid and substantial reduction in bacterial counts within a short timeframe. These findings support the potential applicability of these phages to suppress *S. flexneri* under ex vivo microbiota conditions and support further investigation in more physiologically relevant models.

### 3.6 Whole genome sequencing of PSW32

**Figure 5:**
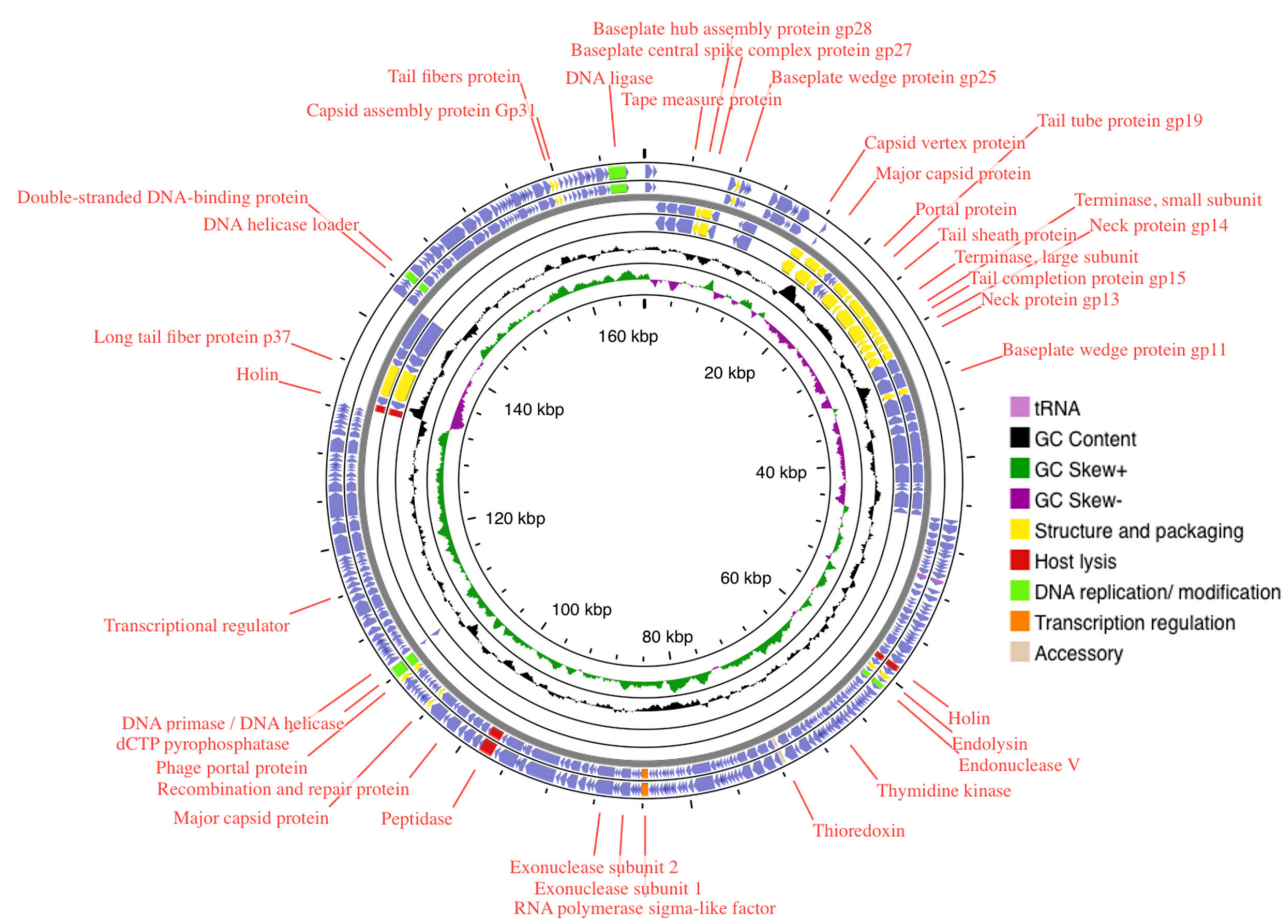
Circular genome map of phage PSW32. The genome was annotated using Bakta and visualized with Proksee (CGView). From the outermost to the innermost rings: Ring 1 shows coding sequences (CDSs) on the forward strand (+), Ring 2 shows CDSs on the reverse strand (–), Ring 3 represents the genome backbone (contig), Ring 4 indicates GC content, and Ring 5 shows GC skew. Genes are color-coded according to predicted functional categories, including structure and packaging, host lysis, DNA replication/modification, transcription regulation, and accessory functions.

The genome of the *Shigella* phage PSW32, shown in (**Figure 5**), was assembled into a single circular contig, representing a large double-stranded DNA phage genome of approximately 167 kb with a GC content of ~40%. Genome annotation using Prokka and Bakta identified numerous coding sequences distributed on both strands and organized into distinct functional modules, including structural and packaging components, DNA replication and modification, transcriptional regulation, host lysis, and accessory functions. The structural module encompassed genes encoding the major capsid protein, portal protein, capsid vertex protein, tail sheath and tail tube proteins, baseplate hub and wedge proteins, tail fiber proteins, and the large and small terminase subunits, consistent with T4-like phage morphology. Genes associated with DNA metabolism, such as DNA polymerase, primase/helicase, ligase, thymidine kinase, endonuclease, and thioredoxin, were also identified, supporting efficient phage replication. BLASTn analysis revealed high nucleotide identity (approximately 96–99.7%) and extensive query coverage (96–99%) with several T4-like *Escherichia* and *Shigella* phages, including *Escherichia* phage CAU_ECP01, LF82-9, CCU7, phiC120, and *Shigella* phage Shf125875, indicating that PSW32 clusters within a T4-like lineage closely related to bacteriophages classified within the genus *Felixounavirus* of the class *Caudoviricetes*, based on genomic relatedness. Whole-genome phylogenetic analysis using VICTOR further corroborated the positioning of PSW32 among T4-like *Escherichia/Shigella* bacteriophages (**Supplementary Figure 1**). In the nucleotide-based phylogenomic tree, PSW32 clustered within a T4-like lineage comprising closely related Enterobacteriaceae-associated bacteriophages, including several phages vB_EcoM_H12, MET_P1_301, HY02, vB_EcoM_ULIM8, and SME50, classified within the genus, while remaining phylogenomically distinct from several previously characterized *Shigella* phage lineages included in the analysis. These findings support the classification of PSW32 based on genomic relatedness rather than solely on morphological characteristics. The phylogenomic differentiation of PSW32 from several previously examined *Shigella* phages highlights the genomic diversity of bacteriophages that infect *Shigella*. The presence of holin and endolysin genes further supports the lytic nature of PSW32. In silico screening using the CARD Resistance Gene Identifier (RGI) implemented in Proksee did not reveal any antimicrobial resistance genes under default parameters. Furthermore, no virulence-linked genes were identified through functional annotation or database comparisons. These findings suggest that PSW32 is a genetically well-characterized lytic phage lacking known resistance or virulence determinants, supporting its potential applicability for future investigation in phage-based applications or biocontrol strategies against *Shigella* spp.

## 4 Discussion

*Shigella* causes significant global morbidity and mortality, and the rapid rise of multidrug-resistant and extensively drug-resistant strains highlights an urgent need for new, non-antibiotic strategies to combat shigellosis. Bacteriophages represent a promising alternative approach due to their potent, host-specific bactericidal activity and advantages over antibiotics, including the ability to target resistant strains while minimizing off-target effects. In this study, we conducted a comprehensive analysis of lytic *Shigella* phages isolated from wastewater sources, examining their morphology, host range, growth dynamics, and antibacterial efficacy, including assessment across multiple MOIs and under simulated gut-like anaerobic conditions, aspects that are often overlooked in previous studies.

Negative-stain transmission electron microscopy (TEM) analysis of the ten isolates revealed a uniform myovirus-like morphology characterized by icosahedral heads and contractile tails (Mallick et al., 2021). This observation is consistent with broader evidence that most known *Shigella* phages belong to the tailed *Caudoviricetes* group (Subramanian et al., 2020). As whole-genome sequencing was exclusively performed for PSW32, the characterization of the remaining phages was therefore limited to morphological analyses. Functionally, the observed lytic activity pattern across 24 *S. flexneri*, 23 *S. sonnei*, 8 *S. dysenteriae*, and 12 *S. boydii* strains corroborated previous findings (Choi et al., 2025), demonstrating relatively broad lytic activity within the genus *Shigella*. The phages demonstrated relatively broad lytic activity against various *S. flexneri* and *S. sonnei* strains, occasional or strain-specific activity against *S. dysenteriae*, and minimal activity toward *S. boydii*. All ten phages demonstrated the capability to lyse every *S. flexneri* strain tested, and most also infected the majority of *S. sonnei* isolates. In contrast, their efficacy against non-*Shigella* enterics was limited. Notably, none of the isolates demonstrated any detectable lytic activity against the tested probiotic strains, further indicating their specificity against *Shigella*. Similarly, another study (Ahamed et al., 2023), exhibited strong activity across diverse *S. flexneri/sonnei* serotypes, with variable susceptibility among *S. dysenteriae*, reinforcing the pattern of relatively broad lytic activity within *Shigella* but narrow activity beyond the genus. While these findings suggest potentially broad lytic activity within the genus *Shigella*, the determination of host range in this study primarily relied on qualitative spot assay. These assays may also reflect lysis-from-without effects, rather than productive infection. Therefore, additional quantitative assessments, such as efficiency of plating (EOP) assays, are necessary to verify productive infection efficiency and delineate the host range more accurately.

Kinetic analysis using one-step growth experiments revealed latent periods ranging from 30 to 70 min, consistent with previously reported values for *Shigella* phages. In contrast, burst sizes exhibited substantial variation, ranging from 27 to 267 PFU/cell. PSW40 exhibited a moderate burst size (81 PFU/cell) and a latent period of 50 minutes, indicating balanced replication dynamics. Notably, PSW32 and PSW39 demonstrated exceptionally high burst sizes, surpassing those reported for phages, such as pSf-1 (Jun et al., 2013) and others, which typically fall within the tens to low hundreds of PFU per cell (Ahamed et al., 2023). These findings suggest that, once adsorbed, several of our isolates exhibit relatively high replication efficiency compared with many *Shigella* phages described in earlier studies. Although the adsorption and washing techniques employed in this study are consistent with standard one-step growth protocols for lytic bacteriophages, it is important to note that adsorption kinetics may vary across different phage-host systems. Furthermore, specific synchronization methods such as low-temperature adsorption or chemical synchronization were not implemented. As a result, variations in adsorption and the initiation of infection among bacterial cells may have influenced the accuracy of the latent period and burst size measurements. Future investigations should incorporate adsorption kinetics assays and advanced synchronization techniques to enhance the precision of replication parameters that are specific to each isolate. Our killing assays, performed across MOIs ranging from 10-0.001, demonstrated near-complete suppression of bacterial growth within 2–5 h depending on the phage tested. Notably, even at the lowest MOI of 0.001, substantial reductions in optical density were observed, indicating strong lytic activity and replication capabilities of these phages. PSW32, PSW36, and PSW39 were the most potent, suppressing bacterial growth within approximately 2.5 h, whereas the remaining isolates required slightly longer. These observations are consistent with previous findings (Ahamed et al., 2019), reported that Sfin-1 effectively suppressed multidrug-resistant *Shigella* spp. at low MOIs, achieving rapid clearance within a few hours of exposure. The comparable rapid killing profiles observed in our isolates suggest that several phages exhibit lytic kinetics comparable to or exceeding those of Sfin-1 under analogous conditions. Importantly, our study extends prior work by systematically evaluating a wider MOI spectrum and incorporating simulated gut-like conditions.

Among the phages exhibiting lytic activity, PSW32 was prioritized for evaluation under ex vivo microbiota conditions based on its broad spot assay-based lytic activity and strong bactericidal properties, making it a suitable candidate for further investigation. PSW40 was additionally included based on its overall lytic performance, including killing efficiency and host range, rather than burst size alone, allowing it to serve as a representative of polyvalent phages with moderate activity under gut-like conditions. Notably, both isolates could infect all four tested *Shigella* species (*S. flexneri, S. sonnei, S. dysenteriae, and S. boydii*) under the spot assay conditions, in contrast to other phages with more restricted spectra. This broad lytic coverage supports further investigation of these phages in *Shigella*-targeted control strategies under microbiota-associated conditions, particularly given the diversity of *Shigella* species and serotypes circulating in community-associated infections. Furthermore, PSW32 and PSW40 differ in burst size and killing kinetics, allowing assessment of whether both high and moderate-performing phages retain efficacy under anaerobic, gut-like conditions. Under ex vivo microbiota conditions, both phages maintained strong antibacterial activity, reducing bacterial levels below the limit of detection (LOD) under the plating conditions used within 3 h. Future studies should investigate the long-term durability of bacterial suppression and assess the potential emergence of phage-insensitive bacterial populations under ex vivo microbiota conditions. One limitation of this study is that pooled donor fecal samples were used to reduce inter-individual variability and provide a generalized gut microbiota environment. However, individual microbiota differences may still influence phage activity and efficacy across hosts, which should be evaluated in future studies. These rapid killing dynamics in a complex gut-like environment align with findings from an ex vivo human tissue study (Llanos-Chea et al., 2019), which demonstrated that phages can inhibit *S. flexneri* adherence to and invasion of human intestinal organoid-derived epithelial monolayers. Moreover, *in vivo* findings demonstrated that orally administered *Shigella* phage SSG23, reduces colonization and shedding in BALB/c mice (Mondal et al., 2025). SSG23 exhibits a latent period of approximately 37 min and yields approximately 195 PFU/cell. While no lytic activity was observed against the non-target strains tested *in vitro*, the impact of these phages on the overall composition of the microbiota was not directly assessed and requires further investigation. Furthermore, this study did not evaluate the stability of bacteriophages in acidic and bile-related gastrointestinal environments. Therefore, the ex vivo microbiota findings should not be considered as evidence of gastrointestinal stability or suitability for oral therapeutic applications.

In contrast, PSW32 demonstrated a comparable or higher burst size and strong lytic activity *in vitro*. Like *Shigella* phage SSG23, PSW32 clusters within a T4-like lineage of large tailed bacteriophages based on whole-genome phylogenetic analysis, while remaining genomically distinct from several other characterized *Shigella* phage lineages. In combination with its broad spot assay-based lytic activity, efficient replication dynamics, and sustained bactericidal activity under ex vivo microbiota conditions, these features indicate that PSW32 has strong lytic activity and represents a promising candidate for future *in vivo* evaluation.

Whole-genome comparative and phylogenomic analyses, including VICTOR-based genome-wide comparisons, placed PSW32 within a T4-like lineage closely related to bacteriophages classified within the genus *Felixounavirus* of the class *Caudoviricetes*, as evidenced through its conserved modular genome structure and strong similarity to previously identified T4-superfamily phages that infect the Enterobacteriaceae family. This architectural conservation, which includes modules for replication, morphogenesis, nucleotide metabolism, and lysis, has been consistently observed in T4-like *Shigella* phages (Zhao et al., 2024). The presence of standard T4 replication-related genes suggests that PSW32 employs a replication strategy typical of virulent myophages, supporting efficient intracellular replication, a trait associated with high lytic productivity in related *Shigella* and *E. coli* phages (Choi et al., 2025). Notably, the absence of lysogeny-related elements and the presence of the holin-endolysin lysis machinery confirm PSW32’s strictly lytic lifestyle, an important feature for potential therapeutic applications. Furthermore, analysis of the PSW32 genome did not reveal any antimicrobial resistance or virulence factors, which is consistent with the genomic safety profiles reported for other anti-*Shigella* T4-like phages (Choi et al., 2025; Zhao et al., 2024). Collectively, these characteristics position PSW32 within the established framework of previously characterized lytic T4-like phages with reported therapeutic relevance, supporting its further evaluation in phage-based applications.

Overall, our findings demonstrate strong *in vitro* and ex vivo bactericidal activity of the isolated phages. Phenotypic characterization indicated that the isolated phages demonstrated common lytic and morphological characteristics typical of *Shigella*-infecting tailed bacteriophages, while genomic analysis of PSW32 specifically supported its placement within a T4-like lineage by revealing a conserved modular architecture, a strictly lytic genetic framework, and the absence of detectable antimicrobial resistance or virulence factors, features consistent with therapeutically relevant virulent myophages. Simultaneously, phenotypic characterization of our isolates expands the existing body of evidence by demonstrating unusually high burst sizes in several candidates, rapid and MOI-robust killing kinetics, and sustained activity under anaerobic gut-like conditions. In particular, PSW32 combines broad spot assay-based lytic activity across *Shigella* species, high progeny productivity, and rapid bacterial reduction in complex media, highlighting its promise for further investigation in phage-based control strategies. These findings further substantiate the potential of bacteriophages as targeted, environmentally favorable, and antibiotic-sparing alternatives for the management of multidrug-resistant *Shigella* infections. Beyond their therapeutic applications, these phages may also hold value in food safety applications targeting *Shigella* contamination. In addition to PSW32, several other candidates (e.g., PSW37 and PSW39) exhibited broad spot assay-based host ranges and strong lytic activity, further underscoring their potential applicability. Continuous monitoring of emerging phage resistance patterns and further *in vivo* validation will be essential for advancing these candidates toward future translational evaluation. Given strain diversity and the risk of resistance development, future studies will incorporate PSW32 alongside other selected phages into rationally designed cocktails to enhance coverage and ensure sustained efficacy. One limitation of this study is that whole-genome sequencing was performed on a single representative phage (PSW32), which restricts the ability to generalize the genomic characteristics and safety profiles to all isolates. In addition, the lack of *in vivo* validation, gastrointestinal stability assessments, and quantitative efficiency of plating (EOP) analyses limits the direct translational interpretation of these findings. Future studies will expand whole-genome sequencing to additional phage isolates to further characterize genomic diversity and confirm safety profiles across the phage collection and evaluate the *in vivo* efficacy and physiological stability of these bacteriophages under gastrointestinal conditions.

## Data Availability

The datasets presented in this study can be found in online repositories. The names of the repository/repositories and accession number(s) can be found in the article/**Supplementary Material**.
