# Supplementary material for: Isolation and characterization of lytic Shigella bacteriophages with rapid in vitro and ex vivo bactericidal activity
Source: Front Cell Infect Microbiol. 2026 Jun 16;16:1837303. doi: 10.3389/fcimb.2026.1837303 (PMC13314873; doi:10.3389/fcimb.2026.1837303)
Supplement: Supplementary file 1 [file Table1.docx]

**Precise Therapeutic Efficacy of Newly Isolated Lytic Phages Against Shigellosis: Rapid Bacterial Activity and Potency in Simulated Gut Models**

Nida Shaheen^1,2^, Maranda Stricklin^3^, Martin Kordesch^4^, Shaohua Wang^1,2*^

^1^Department of Biomedical Sciences, Ohio University Heritage College of Osteopathic Medicine, Ohio University, Athens, OH, 45701 USA

^2^Infectious and Tropical Disease Institute, Ohio University, Athens, OH, 45701 USA

^3^ Department of Biological Sciences, Virginia Polytechnic and State University, Blacksburg, VA, 24061 USA

^4^ Department of Physics and Astronomy, Ohio University, Athens, OH 45701, USA

***Corresponding author:**

Shaohua Wang, Ph.D.,

Department of Biomedical Sciences, Ohio University Heritage College of Osteopathic Medicine, Ohio University

Address: 7 Depot St, Ohio University, Athens, OH, USA 45701

Email: [wangs4@ohio.edu](mailto:wangs4@ohio.edu)

Phone: 1-740-593-2355

Fax: 1-740-597-2778

**Supplementary Table 1.** Morphological measurements of isolated lytic *Shigella* phages.

| **Phages** | **Head (nm)** | **Tail (nm)** |
| --- | --- | --- |
| **PSW29** | 70.8. ± 1.98 | 102.3 ± 1.54 |
| **PSW30** | 68.3 ± 1.67 | 95.4 ± 2.73 |
| **PSW31** | 42.7 ± 1.46 | 53.1 ± 0.29 |
| **PSW32** | 178 ± 2.93 | 188 ± 0.58 |
| **PSW35** | 90.4 ± 3.64 | 181 ± 1.17 |
| **PSW36** | 117 ± 1.06 | 181.3 ± 1.17 |
| **PSW37** | 106.2 ± 1.55 | 172 ± 0.56 |
| **PSW38** | 54.3 ± 1.31 | 73.3 ± 0.40 |
| **PSW39** | 67.2 ± 0.88 | 60.6 ± 0.45 |
| **PSW40** | 166.5 ± 1.48 | 181 ± 0.75 |

**Supplementary Table 2.** One-step growth kinetics of isolated *Shigella* phages

| **Phages** | **Latent period (min)** | **Burst size** |
| --- | --- | --- |
| **PSW29** | 40 | 111 |
| **PSW30** | 70 | 120 |
| **PSW31** | 50 | 91 |
| **PSW32** | 50 | 259 |
| **PSW35** | 50 | 89 |
| **PSW36** | 30 | 40 |
| **PSW37** | 70 | 91 |
| **PSW38** | 60 | 27 |
| **PSW39** | 50 | 267 |
| **PSW40** | 50 | 81 |

**Supplementary Table 3.** Bacterial strains and probiotic isolates used for host range determination and specificity assessment of *Shigella* bacteriophages

| **Bacterial species** | **Number of strains** | **Source/Origin** | **Notes** |
| --- | --- | --- | --- |
| *S. flexneri* | 24 | Brigham and Women’s Hospital (n=2); San Diego County Public Health Laboratory (n=22) | Includes MDR clinical isolates |
| *S. sonnei* | 23 | Brigham and Women’s Hospital (n=3); San Diego County Public Health Laboratory (n=20) | Clinical isolates |
| *S. dysenteriae* | 8 | San Diego County Public Health Laboratory | Clinical isolates |
| *S. boydii* | 12 | San Diego County Public Health Laboratory | Clinical isolates |
| *Escherichia coli*  UPEC-CET073 | 1 | Laboratory stock | Uropathogenic strain |
| *Klebsiella pneumoniae* | 3 | Laboratory isolates | NCBI# PX671225, PX671227, PX671226 |
| Probiotic strains | 11 | Previously isolated and characterized in our laboratory (Gurung et al. 2025) | Used for specificity assessment |

**Supplementary Figure 1.**


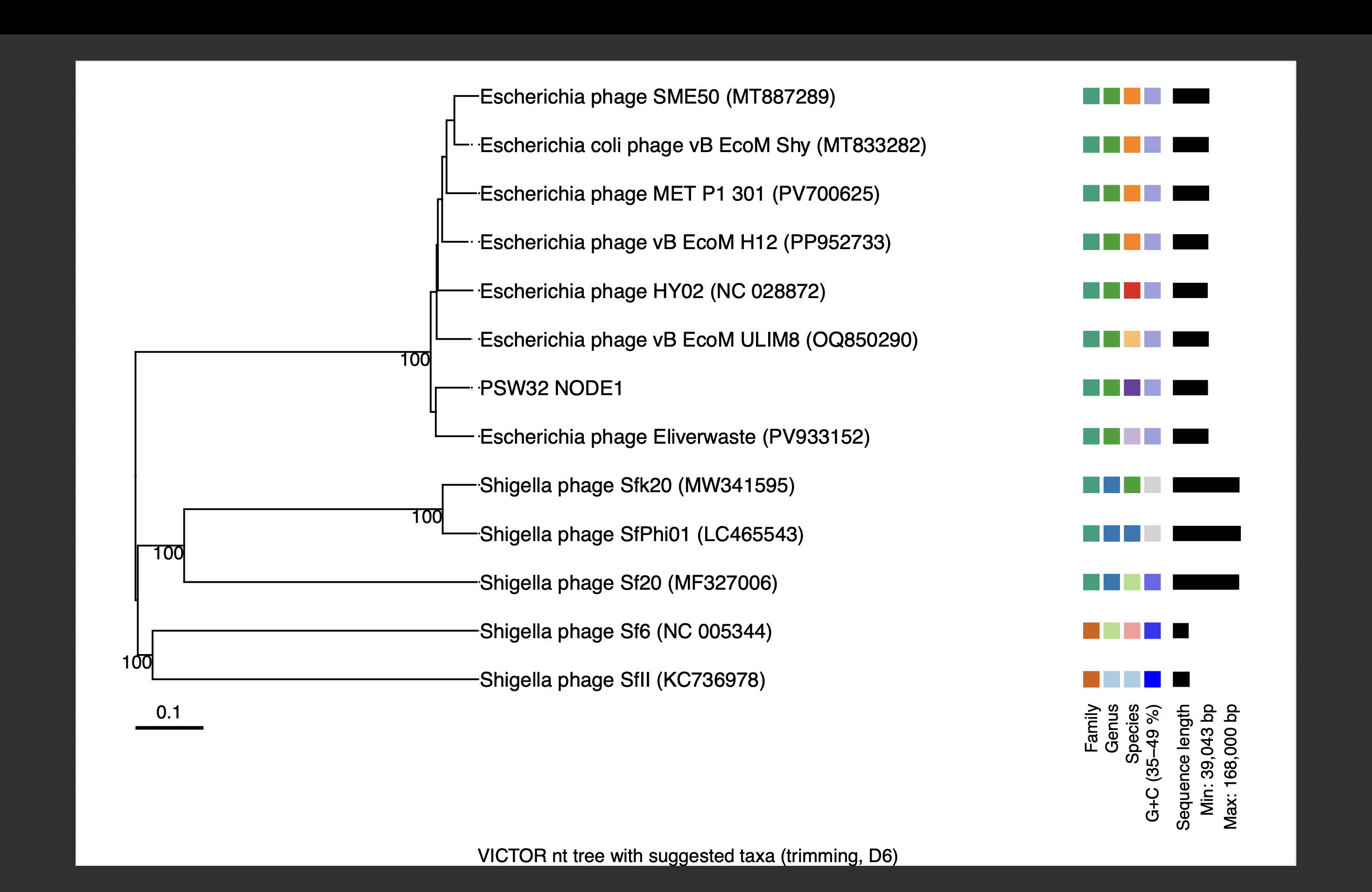


**Supplementary Fig.1.**VICTOR-based whole-genome phylogenetic analysis of bacteriophage PSW32 and related *Escherichia/Shigella* phages. The scale bar denotes the genomic distance.
